# An event-based opto-tactile skin

**DOI:** 10.3389/fnins.2025.1735068

**Published:** 2026-01-23

**Authors:** Mohammadreza Koolani, Simeon Bamford, Petr Trunin, Simon F. Müller-Cleve, Matteo Lo Preti, Fulvio Mastrogiovanni, Lucia Beccai, Chiara Bartolozzi

**Affiliations:** 1 Event-Driven Perception for Robotics, Istituto Italiano di Tecnologia, Genoa, Italy; 2 Soft BioRobotics and Perception Lab, Istituto Italiano di Tecnologia, Genoa, Italy; 3 The Open University Affiliated Research Centre at Istituto Italiano di Tecnologia (ARC@IIT), Istituto Italiano di Tecnologia, Genova, Italy; 4 Advanced Robotics Centre, Department of Mechanical Engineering, National University of Singapore, Singapore, Singapore; 5 TheEngineRoom, Department of Informatics, Bioengineering, Robotics and Systems Engineering, University of Genoa, Genova, Italy

**Keywords:** dynamic vision sensors, event-based sensing, neuromorphic engineering, optical skin, soft robotics, stereo vision, tactile sensing

## Abstract

This paper presents a neuromorphic, event-driven tactile sensing system for soft, large-area skin, based on the Dynamic Vision Sensors (DVS) integrated with a flexible silicone optical waveguide skin. Instead of repetitively scanning embedded photoreceivers, this design uses a stereo vision setup comprising two DVS cameras looking sideways through the skin. Such a design produces events as changes in brightness are detected, and estimates press positions on the 2D skin surface through triangulation, utilizing Density-Based Spatial Clustering of Applications with Noise (DBSCAN) to find the center of mass of contact events resulting from pressing actions. The system is evaluated over a 4,620 mm probed area of the skin using a meander raster scan. Across 95 % of the presses visible to both cameras, the press localization achieved a Root-Mean-Squared Error (RMSE) of 4.66 mm. The results highlight the potential of this approach for wide-area flexible and responsive tactile sensors in soft robotics and interactive environments. Moreover, we examined how the system performs when the amount of event data is strongly reduced. Using stochastic down-sampling, the event stream was reduced to 1/1,024 of its original size. Under this extreme reduction, the average localization error increased only slightly (from 4.66 mm to 9.33 mm), and the system still produced valid press localizations for 85 % of the trials. This reduction in pass rate is expected, as some presses no longer produce enough events to form a reliable cluster for triangulation. These results show that the sensing approach remains functional even with very sparse event data, which is promising for reducing power consumption and computational load in future implementations. The system exhibits a detection latency distribution with a characteristic width of 31 ms.

## 1 Introduction

Soft tactile sensors detect and interpret mechanical stimuli by leveraging compliant materials' inherent flexibility and adaptability. In the past few years, various transduction mechanisms have been extensively explored, including piezoresistive, capacitive, inductive, and optoelectronic approaches (Bartolozzi et al., 2016). Among these, optical sensing stands out due to its wide sensitivity range, excellent reliability, and inherent resistance to electromagnetic interference (Lo Preti et al., 2022a). Unlike many tactile sensors relying on dense wiring or embedded electronics within the sensing area, non-array soft optical waveguides benefit from a sensitive area devoid of wires and rigid parts, delivering fully flexible and adaptable sensing surfaces (Kamiyama et al., 2004; Li et al., 2022). This feature mainly benefits tactile systems requiring conformity to complex or changing surfaces (Chorley et al., 2009; Guo et al., 2022). Moreover, spatial data in optical soft sensors can be retrieved from broad regions without embedded electronics, relying on data processing, which supports seamless integration into robot platforms and versatile applications in environments that are dynamic, unstructured, or subject to frequent change (Guo et al., 2022; Faris et al., 2023).

Two main types of optical tactile sensors can be identified, namely Frustrated Total Internal Reflection (FTIR) and vision-based systems. On the one hand, FTIR sensors operate by detecting changes in light propagation within an optical waveguide caused by deformation or contact, thus allowing a precise measurement of pressure and shape through photodetection (Trunin et al., 2025; Preti et al., 2025). Vision-based optical sensors, on the other hand, utilize cameras to capture deformation or changes in the sensor surface (Shimonomura, 2019). Vision-based optical sensors, which capture deformation or surface changes via cameras, can be divided into frame-based and event-based approaches. Frame-based vision tactile sensors typically capture images at fixed rates to monitor surface deformation or marker displacements, enabling spatially detailed force and shape sensing. For example, Kamiyama et al. (2004) use color-coded markers embedded in a transparent elastic layer to map three-dimensional contact forces, aiding delicate robot tasks. The compact DIGIT sensor (Lambeta et al., 2020) implements low-cost, high-resolution tactile feedback with a modular elastomer design suitable for robust in-hand manipulation and industrial use. Alternative optical methods, such as the retrographic sensor by Johnson and Adelson (2009) leverage photometric stereo to detect surface texture and shape, while Trueeb et al. (2020) employ multiple cameras and adopts Deep Learning (DL) for 3D force reconstruction over larger areas, enabling scalable robot skins. Inspired by human fingertip anatomy, the TacTip sensor (Chorley et al., 2009) uses internal marker displacement within an artificial papillae structure to achieve sensitive edge detection and manipulation without any embedded electronics.

Dynamic Vision Sensors (DVS)—also known as event cameras—are inspired by biological vision systems and detect brightness changes asynchronously, providing high-speed, low-latency, and data-efficient sensing (Lichtsteiner et al., 2008; Gallego et al., 2022). Event-based approaches have driven advances in tactile perception for robots. Event-based approaches have driven advances in data-efficient tactile perception for robots. Recent reviews highlight that neuromorphic tactile sensors emulate biological mechanoreceptors using spike-based, event-driven encoding, producing sparse, low-latency, and energy-efficient touch signals Liu et al. (2025); Kang et al. (2024). For example, Faris et al. (2023) integrate DVS cameras into a soft robotic finger, achieving proprioception and slip detection via real-time event-based heat maps with a 2 ms latency, enhanced by DL techniques and a flexible fin-ray finger design. Similarly, Naeini et al. (2020) demonstrates a successful application of DVS sensors for high-sensitivity force estimation and material classification, highlighting reduced computational and energy demands compared to frame-based methods in dynamic scenarios. The hybrid DAVIS sensor (Brandli et al., 2014), which generates as output both asynchronous events and synchronous frames, is employed by Rigi et al. (2018) with silicone substrates to detect slip and vibration, therefore showcasing suitability for rapid, high-resolution tactile feedback. Building on marker-based tactile sensing, NeuroTac combines the TacTip design (Lepora, 2021) with DVS sensors to enable object recognition, shear detection, grasp stabilization, and manipulation for anthropomorphic robots Ward-Cherrier et al. (2020). In a complementary approach, (Sferrazza and D'Andrea 2019) developed a high-resolution optical sensor using marker tracking and DL for real-time force prediction on soft surfaces. More recently, Funk et al. (2024) introduced Evetac, an event-based optical tactile sensor leveraging 1,000 h event readout rate to sense vibrations up to 48 h, reconstruct shear forces, drastically reduce data rates versus RGB sensors, and demonstrate data-driven slip detection within a robust closed-loop grasp controller. In a complementary approach, Sankar et al. (2025) demonstrate a biomimetic prosthetic hand with three layers of neuromorphic tactile sensors, enabling compliant grasping and achieving ≈99.7% accuracy in texture discrimination.

The approaches mentioned above focus on small-scale sensors for fingertips, with limited examples of large-area tactile skins. Additionally, many of these solutions rely on high-resolution vision sensors, which often require substantial computational resources and the use of advanced data-driven techniques, such as Deep Neural Networkss (DNNs) or Reinforcement Learning (RL), to process the vast amount of generated data. While promising, these approaches may face scalability, energy efficiency, and real-time processing challenges, especially for large-scale, adaptive soft robot systems. As an extreme example of multimodal touch sensing, Lambeta et al. (2024) built an artificial fingertip with approximately 8.3 million taxels capable of capturing fine pressures, high-frequency vibrations (up to 10 kh), thermal cues, and chemical traces, all processed by on-chip AI.

To the best of our knowledge, we present the first system integrating stereo DVS sensors with an optical skin to perform contact localization via triangulation. Unlike prior works mostly emphasizing force estimation on small fingertip areas, our approach provides a lightweight solution for the localization of contacts, scalable to large flexible skin areas, using minimal computation without relying on either DNNs or complex marker tracking. However, contact-point localization has also been studied acoustically, for example, Lee et al. (2024) introduced “SonicBoom,” in which a distributed microphone array mounted on a robot link pinpoints single taps with sub-centimeter accuracy even under occlusions.

This study builds on Lo Preti et al. (2022b)'s Multitouch Soft Optical Waveguide Skin (MSOWS), a marker-free, optical skin that achieves large-area tactile sensing without embedded electronics in the sensing area. The design combines graded-stiffness Polydimethylsiloxane (PDMS) layers and a Near-infrared (NIR) optical system to sense pressure, providing adaptability to complex shapes and durability against repeated mechanical stress. MSOWS uses pulsed light sources and repetitive sequential scanning through photodiodes integrated around the skin perimeter. The rate of this scan introduces inherent latency into the detection process, which can only be reduced by increasing the scan rate and therefore the volume of generated data. In this work, we leverage the low-latency change detection capabilities of event-driven photoreceptors to detect the changes in illumination due to tactile contacts on the silicone surface. As a first step to substitute the array of photodiodes in Lo Preti et al. (2022b), we conducted a preliminary study using a pair of DVS integrated in the silicone layer for contact localization.

As a potential skin covering, applicable to larger areas, this setup allows for an efficient triangulation and spatial analysis of touch positions, therefore contributing to the development of scalable and adaptive tactile sensors in soft robots. Unlike the above-mentioned approaches, which rely on DNNs or heavy data-driven algorithms, this work uses a simple, lightweight triangulation for localizing pressure locations on the silicone surface, which is crucial for real-time applications.

An overview of the performance metrics of State of the Art (SOTA) optical tactile sensors is summarized in Table 1, comparing with our approach and showing their hardware, the measurements, the algorithms, and the task-relative metrics.

**Table 1:** Comparison of this work and similar tactile sensors.

**Sensor**	**Hardware**	**Primitive**	**Algorithm**	**Metrics**
Force-EvT (Guo et al., 2024)	DVS	Force estimation	Vision transform	RMSE 0.13 n (Newtons) R^2^ = 0.3 1.5 % error
Vision-based (Naeini et al., 2020)	DVS	Contact force	Time delay neural network	RMSE 0.16–0.17 n 7.2 % accuracy
Bio-inspired (Faris et al., 2024)	DVS	Slip & Pressure	Spike processing	5 % accuracy 2 ms RT
Evetac (Funk et al., 2024)	DVS	Vibration & Shear force	DL	Vibration sensing up to 48 h
E-BTS (Mukashev et al., 2025)	DVS	Force estimation	DL force prediction	RMSE ~1 n Latency < 5 ms
NeuroTac (Ward-Cherrier et al., 2020)	DVS	Texture/object recognition, slip cues	Spiking neural network (event-based)	Classification accuracy (task-dependent; e.g., 95% on Braille)
Multi-cam (Trueeb et al., 2020)	4 RGB cameras with silicone	Force distribution	Deep Convolutional Neural Network (DCNN)	RMSE 0.057 n (Fz) 40 h
DIGIT (Lambeta et al., 2020)	RGB camera with elastomer	Shape & force	Autoencoder	640 × 480 px @ 60 h
Retrographic (Johnson and Adelson, 2009)	RGB with elastomer	Texture/shape	Photometric stereo	High-res 2.5D capture
MSOWS (Lo Preti et al., 2022b)	LED with photoreceiver	Localization	Sequential scan	5 mm resolution 2.6 % error
This work	Stereo DVS	Contact location	Stereo with DBSCAN	RMSE 4.66 mm 31 ms detection latency distribution

## 2 Materials and methods

### 2.1 Sensor design and manufacturing process

This paper introduces a new design for a vision-based sensor using two DVS cameras, partly inspired by classic soft optical sensors integrating LEDs and receivers within soft, transparent silicone substrates. This sensor features a square 100 mm per side and 4 mm thick silicone layer, embedding two DVS cameras (Prophesee EVK1 VGA Gen3.1 sensor, resolution: *u* = 640, *v* = 480), positioned at the corners of one edge. Furthermore, 32 NIR LEDs are mounted along the remaining three edges, all pointing to the center of the edge with the two cameras. This setup helps enhance the visibility of the pressure distribution applied to the silicone layer. The LEDs emit light into the elastic silicone element, where deformation within the material affects the light intensity, which is then detected by the DVS cameras. Using two cameras allows for stereopsis, therefore enabling a 2D localization of pressure locations on the skin.

**Figure 1:**
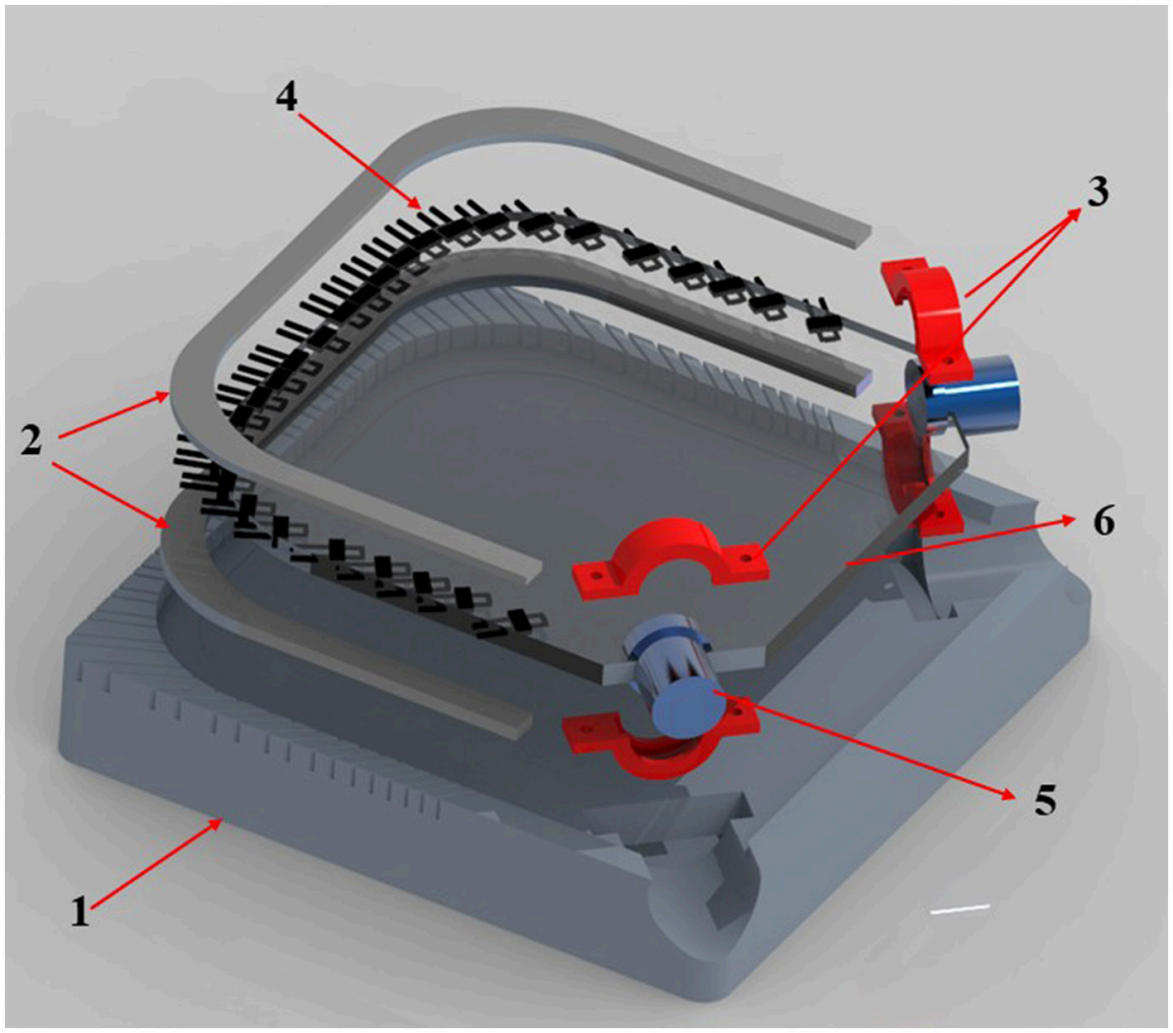
Exploded view of the sensor design. Components: (1) ABS printed base; (2) emitter mounts; (3) lens mounts; (4) near-infrared VSMY1860 emitters; (5) ABS lens replicas used during molding; (6) PDMS tactile layer.

The sensor design is shown in Figure 1. The base (1), printed from Acrylonitrile Butadiene Styrene (ABS) on an Ultimaker S3 (Ultimaker, Utrecht, NE), houses mounts for the emitters (2) and camera lenses (3). NIR VSMY1860 emitters (4) and ABS-printed lens replicas (5) are placed into these mounts. 3D-printed dummy lenses were used in the silicone casting process, allowing for easy removal without damaging the silicone layer. Those replicas are then replaced with actual lenses afterward.

After assembling all components, silicone (6) was prepared and deposited in the mold. Due to its well-known optical properties, particularly its optical transmittance, PDMS was chosen as the elastic element. A 30:1 ratio of PDMS base to curing agent was prepared and mixed using a Thinky Mixer (THINKY U.S.A., INC.), then degassed in a vacuum chamber for 7 m before being poured into the mold. This ratio was chosen to make the PDMS as soft as possible, enhancing the sensor's sensitivity and flexibility (Sales et al., 2021). This softer composition facilitates the detection of subtle interactions and deformations, improving its responsiveness and the overall performance. Finally, the sensor was cured in an oven for 2 h at 75 c.

### 2.2 Experimental setup and data acquisition

**Figure 2:**
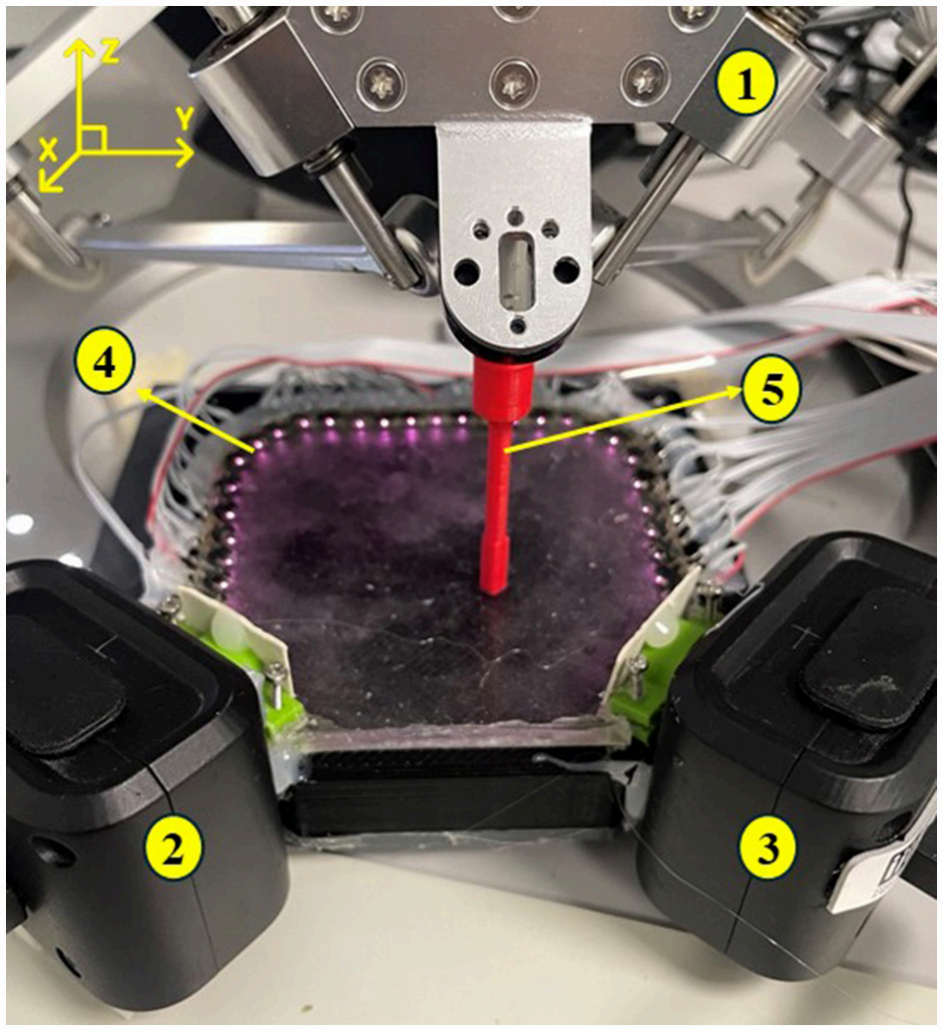
Experimental setup for tactile data acquisition with our optical skin system. The setup includes DVS cameras (2 and 3) placed on two adjacent sides of a rectangular silicone layer to capture dynamic touch events. The Omega 3 force dimension robot (1) applies a controlled force using the tip (5) to predefined points on the silicone surface, creating deformations detected by the cameras. NIR LEDs (4) provide consistent illumination, allowing detection of surface deformations.

Data acquisition in the setup shown in Figure 2 is carried out by applying an external force to the silicone layer using an Omega 3 Force Dimension robot. This leads to deformation and brightness changes detectable by the DVS cameras. The sensing area is rectangular, with DVS cameras at two corners. This arrangement covers most of the tactile sensing area, allowing each camera to capture variations in brightness caused by the deformation of the silicone layer illuminated by NIR LEDs placed on the other sides, as illustrated in Figure 1. The NIR LEDs (16 mw total power consumption) provide consistent illumination, thus ensuring high visibility of the silicone layer even in low-light conditions, enhancing the accuracy of touch-point detection. This configuration is expected to support efficient and highly dynamic range data acquisition, enabling responsive and adaptable sensing in robots and haptic settings.

The sensor was pressed at 250 locations arranged in a meander path with 4 mm spacing between each point at a constant depth of 2 mm (it should be mentioned that the applied force was not analyzed). To ensure data consistency and robustness, the grid pressing pattern was repeated 10 times, each involving the same 250 pressing points. After completing each set of 250 presses, the robot returns to the starting position to begin the next repetition.

To ensure temporal alignment between the two DVS recordings, a synchronization procedure is performed prior to the main experiment. Since the recordings were started manually and were not inherently synchronized, the center of the skin—clearly visible to both cameras—was pressed three times with 1-s intervals, followed by a 3-s pause before the first actual press. These three initial press actions serve as temporal landmarks, enabling a precise alignment of timestamps across both camera streams. The aligned event sequences ensure the accurate segmentation and a consistent comparison of press timings. These initial calibration actions are highlighted in Figure 4A with a rectangle.

**Figure 3:**
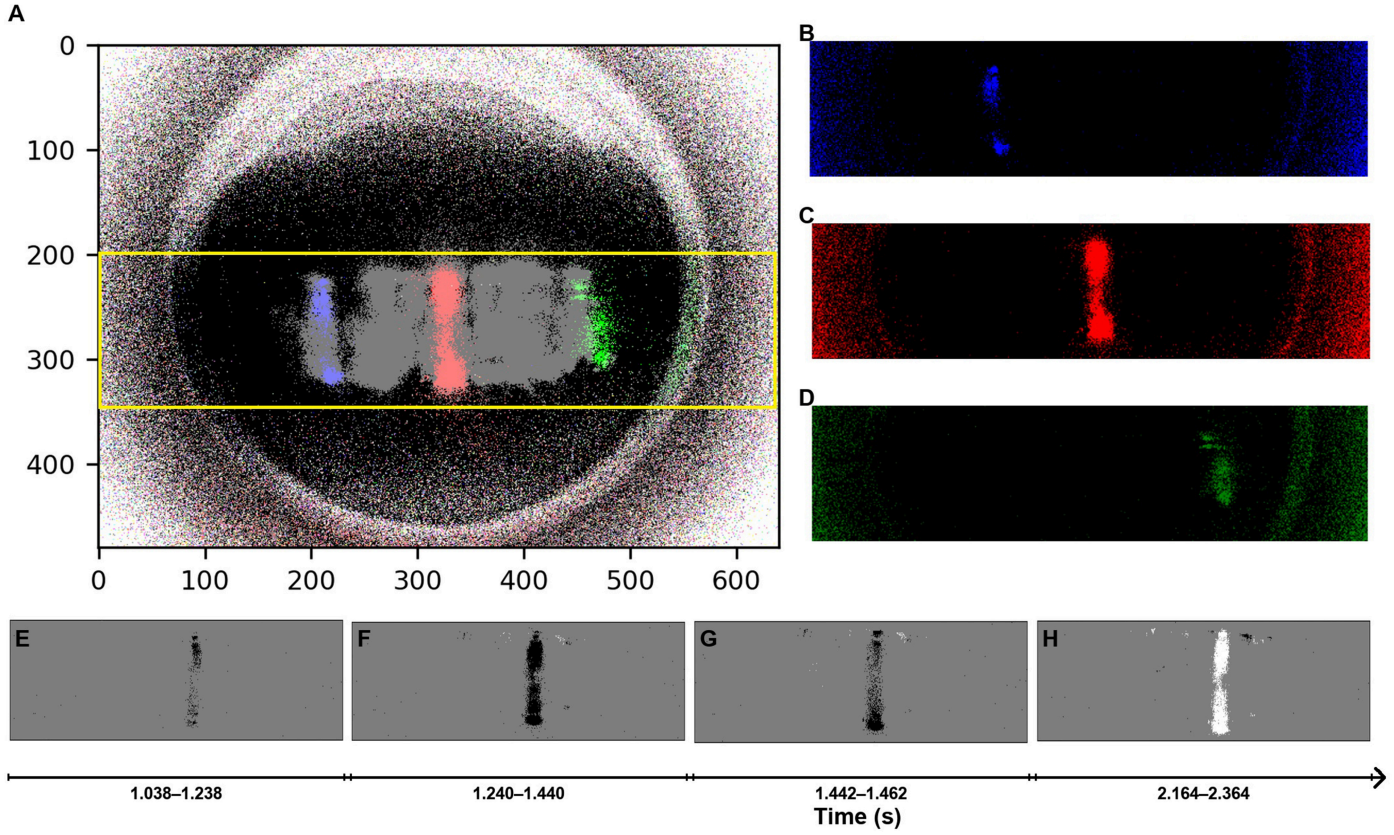
Overview of DVS camera event data illustrating press actions localization based on the circular path. **(A)** Shows the accumulated event rates across all contacts, providing a high-level view of spatial activity distribution over the sensing area. The yellow box indicates the cropped region (v = 200–360), and only the pixels inside this box are retained for analysis. **(B–D)** Represent individual contacts captured by the same camera, each shown in a color-coded channel. These images demonstrate the sensor's sensitivity to different press locations, as each press generates a unique pattern of event distribution. **(E–H)** (right column) display a temporal sequence for a single contact, highlighting spatiotemporal characteristics that could be useful for further classification.

**Figure 4:**
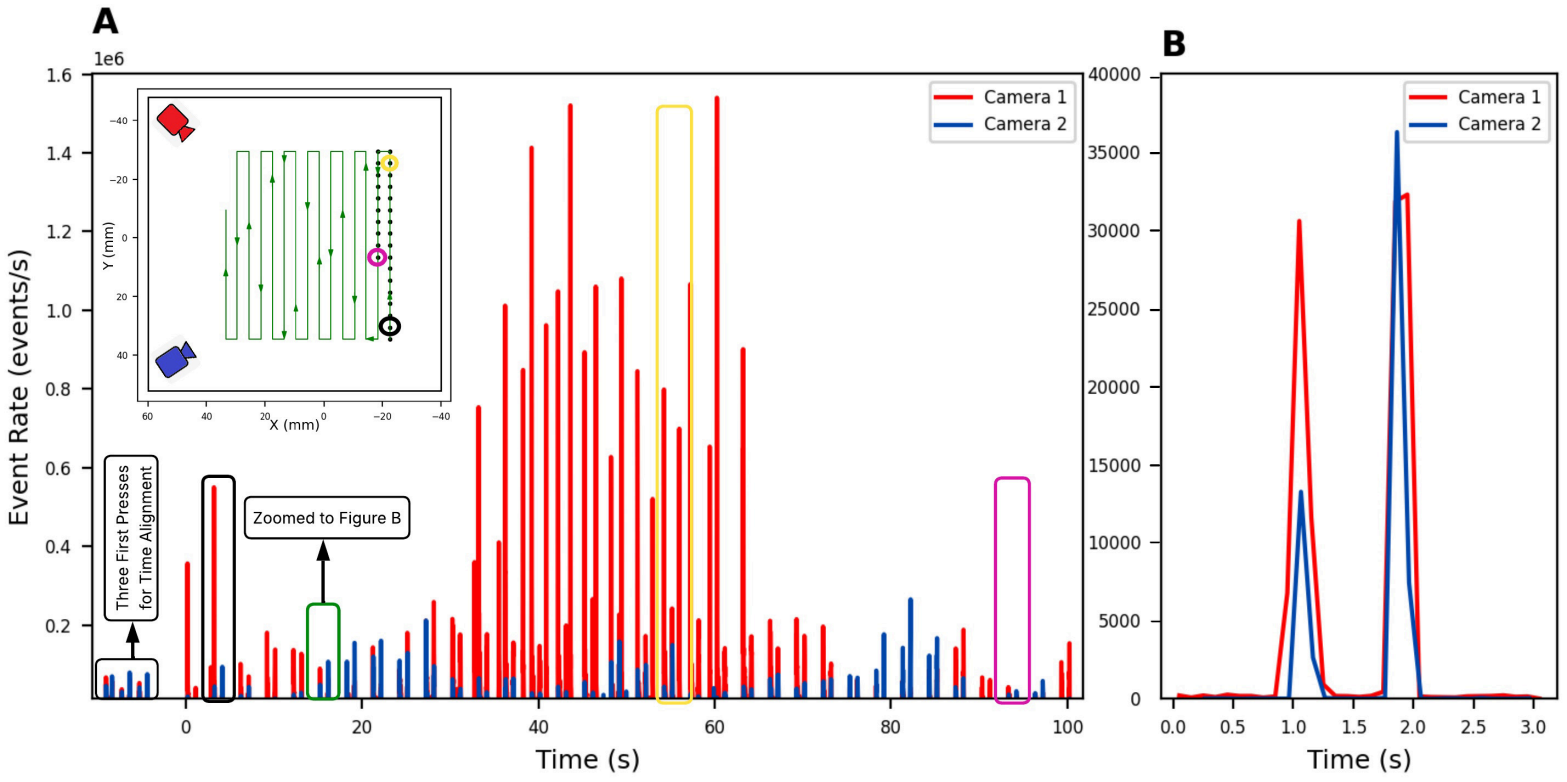
Event rate analysis during skin pressing, computed using histograms with a bin size of 10 ms. **(A)** Event rate over the first 100 s of activity from Camera 1 (red) and Camera 2 (blue), corresponding to the first 27 presses performed in a meandering path. The inset shows the sensor area and pressing path, with camera positions indicated (red and blue) and individual presses marked. Selected presses are highlighted with color-coded rectangles on the event-rate plot and linked to circles of the same color on the sensor map: green for the 2nd press, yellow for the 16th press, and purple for the 27th press. **(B)** Event-rate for a single pressure instance recorded by both cameras, overlaid to show synchronization and comparative sensitivity. Camera 1 is plotted in red and Camera 2 in blue consistently.

**Figure 5:**
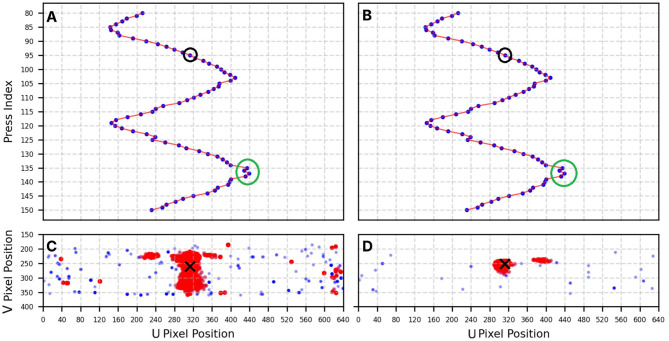
The spatial distribution of pixel activity using DBSCAN. This figure visualizes the estimated activity centers across the sensor surface based on DBSCAN clustering of pixel events. **(A, B)** show the top-down views of the *u*−coordinate centroids extracted from each press for Camera 1 and Camera 2, respectively. These views illustrate the spatial spread of detected presses along the circular path. **(C, D)** show example contact activity for Camera 1 and Camera 2, where the DBSCAN-identified pixel clusters have been processed to extract their centroids, representing the dominant activity locations. In these examples, the clusters correspond to press number 95, which is marked with a black circle in **(A, B)**, and the black cross in **(C, D)** indicates the center of the activity for that press.

The accumulated event rates for contacts on the circular path (48 presses on two circles with radii of 20 mm and 25 mm) are shown in Figure 3A. This dataset is used solely to illustrate how press-induced event activity appears on the sensor surface, as the circular pattern provides visually clear and evenly distributed examples of event generation. No localization analysis is performed on this dataset. All quantitative results presented in this work—including triangulation accuracy, pass-rate analysis, and detection latency distribution evaluation—are based exclusively on the meandering-path dataset shown in Figures 4, 5.

Much of the sensing area remains unused, as seen by the outer ring around the viewable area. The images in Figures 3B–D represent individual contacts on the same camera, each shown in a different color. These images highlight the varying activity levels across different pressure locations, illustrating how each press action generates a distinct pattern of event distribution on the sensor.

Each contact captured by the DVS cameras exhibits unique spatio-temporal characteristics, as illustrated in the sequence of images in Figures 3E–H on the right, which show the stream of events generated from a single press with events accumulated over a fixed time window. In these images, gray pixels represent areas without events, while black and white pixels indicate events with negative and positive polarities, respectively, corresponding to decreases and increases in light intensity detected by the sensor. This reflects how light from LEDs is blocked or revealed during skin deformation as the indenter presses and releases. As illustrated in Figure 2, the cameras are embedded at the edge of the skin, looking through the side of the silicone. The events are generated by the indenter pressing the top surface of the skin, causing deformations and interrupting the light coming from the LEDs visible from both cameras. This temporal progression of events highlights changes in the activity and could be leveraged for further processing, extracting additional information from the data, such as the classification of contact types, slip detection, or motion patterns. However, this study is focused on the localization of contacts based on aggregated event statistics.

### 2.3 Data processing and press actions segmentation

The DVS cameras generate continuous event data, with each press action on the optical skin generating a series of events. Initial data processing begins by calculating the event rate (number of events per second) across all press actions, offering a high-level overview of tactile activity over time. Figure 4A displays the event rate from both Camera 1 (red) and Camera 2 (blue) over the first 100 s of the experiment. This corresponds to 27 individual pressures applied in a meandering path, as illustrated in the inset on the top left. The inset also shows the spatial layout of the sensor and the robot's pressing path, with the relative positions of the two cameras (red and blue) and the pressure trajectory over the optical skin. Each contact location is marked, and selected pressures are connected to their corresponding spikes in the event-rate plot using green rectangles for visual reference.

To remove irrelevant activity and focus only on tactile interactions, the upper and lower part of the visual field was cropped (on the v-axis, from *v* = 200 to *v* = 360), based on the 4 mm thickness of the silicone layer and the lens characteristics. The cropped region corresponds to the yellow box shown in Figure 3A.

Furthermore, to enable press-wise analysis, event data were segmented temporally into individual pressure events. Figure 4B zooms in on a single contact and shows the corresponding event-rate for Camera 1 (red) and Camera 2 (blue), highlighting synchronization and sensitivity of both views during a single tactile event.

### 2.4 Event detection and finding the centroids

After segmenting the individual pressures, we used the clustering-based approach Density-Based Spatial Clustering of Applications with Noise (DBSCAN) to find the horizontal centroid of each contact on the sensor of each camera.

DBSCAN identifies the density of the clusters of contact activity without prior assumptions about the number of events or the shape of the clusters. As all presses are separated into individual containers based on their timing, DBSCAN can be applied to spatial data to find the region with maximum activity. Events that do not belong to any cluster are excluded as noise. DBSCAN was applied with an ε (eps) neighborhood of 10 pixels and a minimum of 10 events per cluster (*min*_*samples* = 10). These parameters were chosen empirically to balance noise rejection with robust cluster formation across different presses.

When one or more clusters are found, the largest cluster is computed and considered as the direction of the contact activity on the sensor's surface. This method filters out scattered noise events and focuses on the activity elicited by pressure contacts. From the remaining events, the centroid of the dominant cluster is computed as the mean of the (*u, v*) positions within the cluster. This centroid is used to represent the pressure location in the image space. Although both *u*- and *v*-coordinates are used for clustering, only the *u*-coordinates of the resulting centroids are retained for subsequent triangulation Section 2.6. This is because the two cameras are arranged along a horizontal stereo baseline, meaning that disparity, and consequently depth information, is primarily encoded in the horizontal (*u*) direction. The *v*-coordinate variations do not contribute to the triangulation accuracy in this configuration but remain useful for verifying the spatial consistency of the detected contact regions. The process is repeated for all presses across both cameras.

Figures 5A, B present the top-down distribution of centroid *u*−positions identified by DBSCAN for contacts numbered from 80 to 150, visualized from the perspectives of Camera 1 and Camera 2, respectively. This visualization offers a mapping of detected pressure locations across the sensor surface. Additionally, Figures 5C, D show individual contact activity distributions with their clustered activity regions.

### 2.5 Data filtering and pressures exclusion criteria

Figure 5 illustrates scenarios in which press localization may fail due to weak or ambiguous event patterns in either view. Such weak pressure activities are highlighted by green circles in Figures 5A, B. To ensure a robust localization, a press was removed if DBSCAN failed to identify a prominent cluster of activity in one of the views. This happens when there is not enough density or structure in the spatial arrangement of events to create a significant cluster, which might indicate that the pressure was too weak or was obscured. A threshold number of cluster points was introduced as a metric to determine the existence of a valid pressure event. To perform the optimization in Section 2.6, some outliers were manually excluded (this is consistent with a one-off calibration process for a new sensor). However, the outliers remaining after thresholding were included.

### 2.6 Geometric triangulation for pressure localization

Once the area of maximum activity for the *u*−coordinate of each camera is found, the angle between the center pixel of each camera's field of view and the identified active pixel (notated as θ_1_ for Camera 1 and θ_2_ for Camera 2) is computed. These angles, along with the known distances (that is, *d*_1_ and *d*_2_) from each camera to the center of the silicone layer, allow us to draw triangulation lines from both cameras toward the detected pressure location. Angles and distances are inputs for geometric triangulation, providing the basis to estimate the press location on the sensor's *x*− and *y*−coordinates.

**Figure 6:**
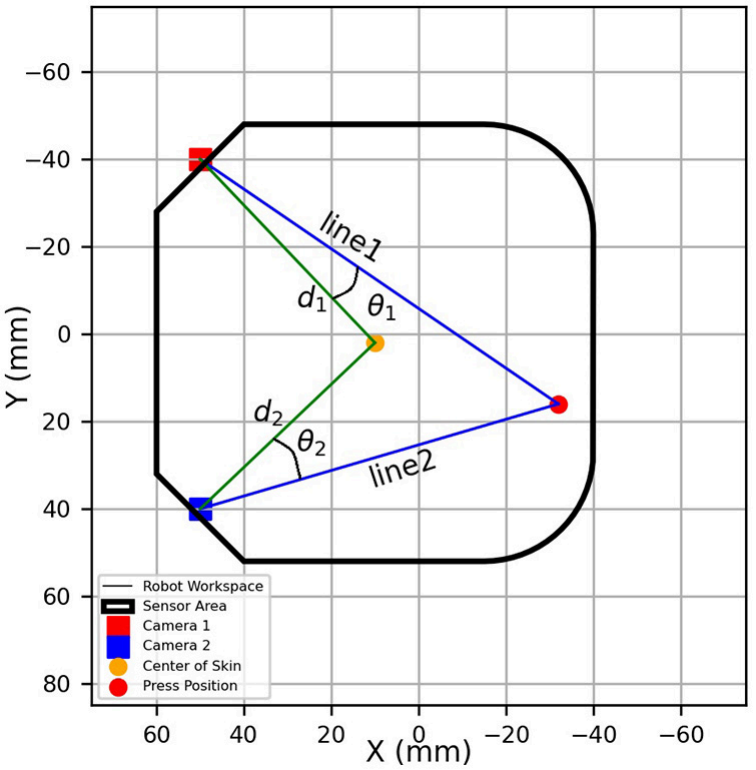
The triangulation process for contact position estimation. This figure shows the triangulation process, where angles (θ_1_ and θ_2_) are calculated between each camera's center and active pixels. Distances (*d*_1_ and *d*_2_) from the cameras to the center of the sensing area are used to draw lines toward the detected pressure position. The intersection of these lines provides the estimated coordinates of the press on the optical skin.

The final pressure position is determined by computing the intersection of the triangulation lines from each camera, representing the estimated coordinates (*x, y*) of the press on the optical skin. DBSCAN allows for the estimation of the activity center, effectively mitigating noise and identifying center regions of pixel activity. Figure 6 illustrates this triangulation process, detailing the angles and distances of locating each press in the sensing area. Although initial triangulation was performed using camera positions measured directly from the robot workspace, further refinement was introduced to improve localization accuracy by treating the following system parameters as optimization variables. Skew angles were introduced into the triangulation model to account for any possible misalignment between the cameras and the edges of the tactile surface. Recognizing that the cameras might not be perfectly parallel to the skin's edges due to setup imperfections, the skew angles and camera positions are treated as variables within the triangulation process. By incorporating skew angles (θ_skew_1__ for Camera 1 and θ_skew_2__ for Camera 2) and optimizing camera positions (*x*_1_, *y*_1_ for Camera 1 and *x*_2_, *y*_2_ for Camera 2) and lens distortion, the accuracy of estimated press locations was improved. The least squares optimization was then performed on these parameters to minimize the difference between the estimated pressure locations and the coordinates provided by the Omega 3 force dimension robot. This optimization was performed for the grid press trajectories to account for any differences in the data patterns. Optimized camera positions, skew angles, and distortion correction improved accuracy, as reflected in the error metrics presented in Section 3.1. It should be noted that, as described in the experimental setup, the grid press path consisting of 250 locations was repeated ten times. The triangulation parameters were optimized using data from one repetition of the path, and the optimized parameters were then applied to the remaining nine repetitions. This approach was adopted to minimize overfitting and to provide a more reliable estimate of the system's localization accuracy.

**Figure 7:**
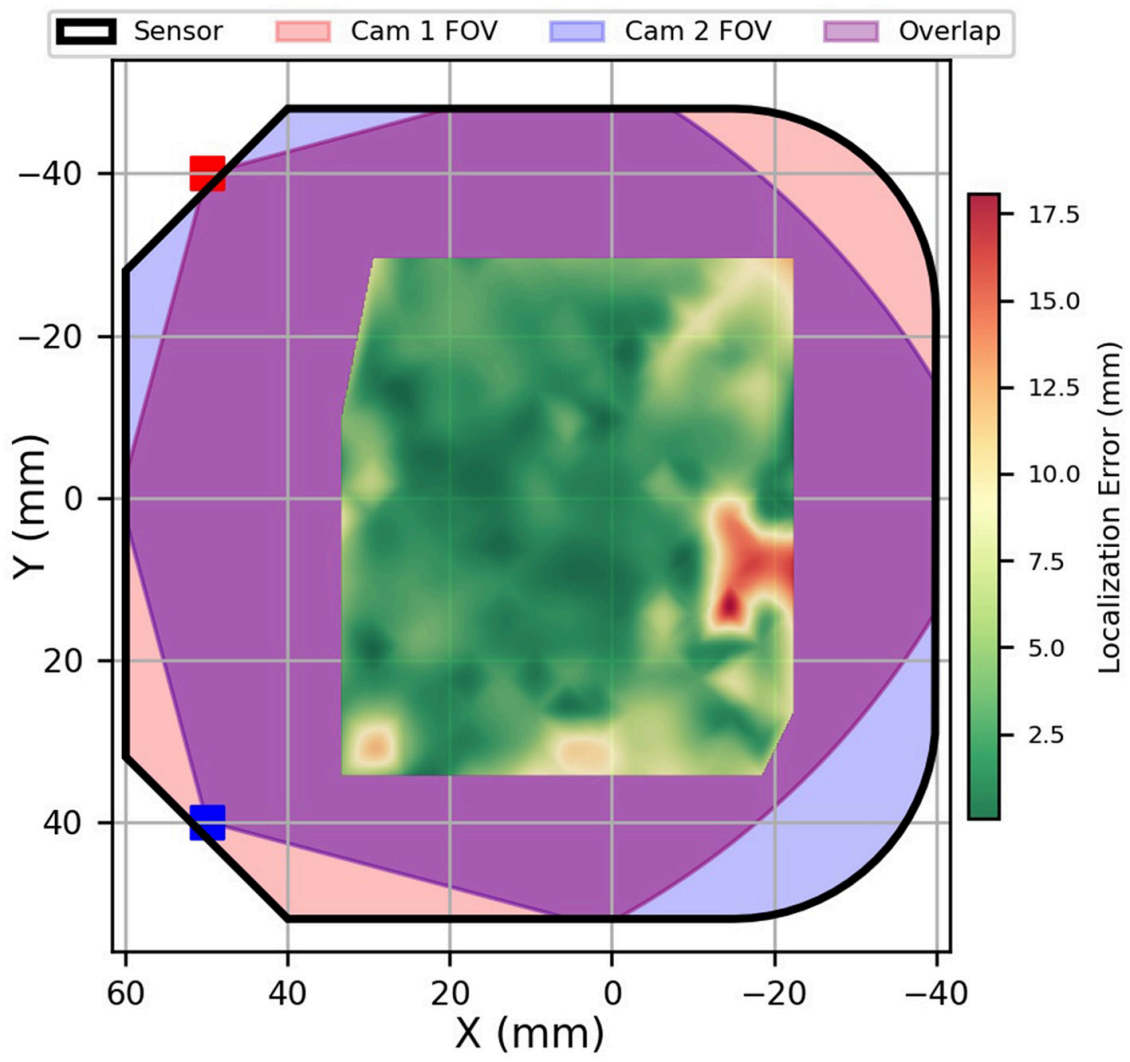
Localization error heatmap for the DBSCAN-based triangulation method on the grid path. Red regions indicate higher error magnitudes, while green regions correspond to more accurate estimations. The field of view (FOV) of each camera, their overlap region, and the sensor boundary are also shown.

The differences in the optimized camera positions for the grid path are visible in Figure 7, where the camera positions are depicted differently for the path. Triangulation calculations are based on these introduced and optimized variables.

## 3 Results

This Section presents the performance of the proposed event-based optical skin in localizing contact events. We report the system's localization accuracy based on ground truth comparisons, analyze its spatial coverage and detection robustness, and evaluate its response to data reduction through ablation testing. In addition, the potential latency distribution of tactile event detection is quantified to assess the sensor's suitability for real-time applications.

### 3.1 Error calculation and comparison

To evaluate the accuracy of the triangulation method, the estimated pressure locations are compared to the ground-truth coordinates provided by the Omega 3 force dimension robot. The Root-Mean-Squared Errors (RMSE) was selected as the primary performance indicator among the calculated error metrics. The RMSE for the grid path is 4.66 mm, which corresponds to 3.75 % of the sensor's diagonal. The mean standard deviation between trials for single pressures on the grid path is 2.85 mm. This indicates that the triangulation approach can estimate press positions, providing sufficient accuracy for practical tactile sensing applications. Figure 7 illustrates the estimated pressure locations derived from DBSCAN for the grid path. To better understand how the localization error is distributed, we also calculated the RMSE separately along the *x*- and *y*-coordinates. The RMSE in the *x*-direction is 4.17 mm, and the RMSE in the *y*-direction is 3.28 mm. These values show how the triangulation geometry contributes differently along each axis and complement the overall Euclidean RMSE of 4.66 mm.

The total area of the fabricated skin surface is 9,328 mm. Out of this area, 8,763 mm (93.9 %) is within the field of view of both cameras. The robot probed 4,620 mm (52.7 %) of the visible skin area. After applying a manual threshold to reject poor quality results, the proportion of probed locations, which are visible to both cameras, where pressures can be reliably detected and localized, is 95 %.

### 3.2 Data ablation and latency distribution analysis

One of the main motivations for this work is achieving a low-data sensor maintaining the accuracy of the localization. High-resolution DVS cameras are utilized in the present realization. Subsequent system versions could use dedicated event sensors with substantially fewer pixels, consequently reducing data transmission and processing demands. Furthermore, optimization of sensor tuning would also enhance responsiveness, with better control over the event rate.

To investigate how much the system depends on the amount of event data, we performed a data ablation (event reduction) analysis. Event data was progressively reduced using predefined downsampling factors (2^0^ to 2^10^), and the localization accuracy was evaluated using the RMSE and pass rate metrics.

The downsampling was done by applying a simple stochastic thinning of the event stream. For a given reduction factor *k*, each event was kept with probability 1/*k* and discarded otherwise. This keeps the original timing of the events but reduces their total number by roughly a factor of *k*. For each reduction factor, we repeated the thinning process using different random seeds to check the consistency of the results.

For each press, we computed the Euclidean distance between the estimated and actual press coordinates. For a given reduction factor, the distribution of these errors was obtained over all presses. A press was counted as a “pass” if its localization error was smaller than the 95^*th*^ percentile of that error distribution. The pass rate is the proportion of presses that meet this criterion. This gives an intuitive measure of how often the system still produces a usable localization after reducing the number of events.

**Figure 8:**
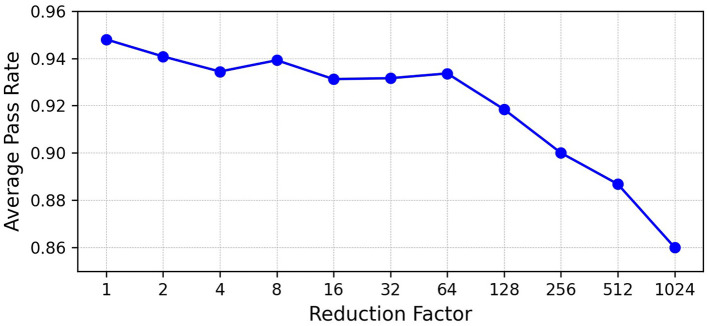
Pass rate trend with event reduction. The system maintains a high detection success rate, even with a reduction factor of 1,024×.

Figure 8 shows the pass rate as a function of the reduction factor. The pass rate stays above 85% for reduction factors up to 2^10^ (1,024), but drops beyond this point, indicating that too much event removal starts to eliminate essential information for localization.

DVS are inherently fast due to their asynchronous event-driven nature, allowing near-instantaneous response to changes in the visual field (Delbruck et al., 2008; Posch et al., 2014). However, we evaluated the potential latency of this system in detecting a press by analyzing event-rate distributions over time, to help understand whether the low-latency of the DVS can be exploited.

**Figure 9:**
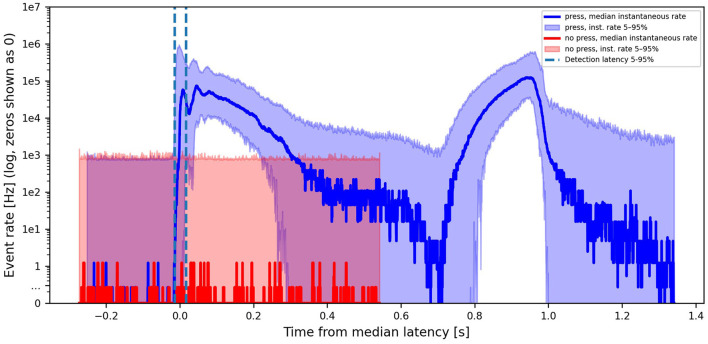
Instantaneous event rates, aggregated over both cameras and all pixels, per press trial (blue) versus background event rate (red), giving median and 5–95% bounds. The dashed vertical lines indicate the detection latency distribution of 31 ms.

Although the robot triggers were regular at known intervals, no hardware-recorded ground-truth onset is available. Therefore, we quantified detection latency distribution by finding the times when the aggregate event rates rise above normal noise and reporting the interval in which most of these crossings occur. For each trial, events were binned at high temporal resolution (0.2 ms bins) and smoothed with a short Gaussian kernel (σ = 0.5 ms). The baseline firing rate was estimated from a short background snippet immediately preceding the stimulus. Onset was then defined as the first threshold crossing of a one-sided Cumulative Sum (CUSUM) detector tuned to a four-fold rate increase over baseline, requiring at least three consecutive bins above threshold. A threshold parameter *h* was optimized by Receiver Operating Characteristic (ROC) analysis to ensure ≥95% of trials were detected within a ±100 ms window of the population median onset, while minimizing the false-alarm rate background snippets.^1^ Figure 9 shows 95th, 50th, and 5th percentile event rates for press versus no-press, with time aligned to the median onset time during presses (*h*=1). The detection latency distribution, i.e., the time between 5th and 95th percentile onset times for true positives, is 31 ms and the false-alarm rate is 0.13 events/s (where the 31 ms is used as a cool-down between consecutive false alarms).

This window is likely an overestimate of the latency from event production to the possibility of contact detection because the method only looks at event rates combined from both cameras and does not consider any other spatiotemporal cues, which could improve detection. At the same time, it underestimates the end-to-end system latency, since any delay from the robotic trigger to the production of the first press-related events is not included. Furthermore, this method is an analysis of the possibility of contact detection based on event statistics, rather than a proposed online algorithm. All of our processing in this work has been offline and trial-based and we have not presented an online method for press detection, therefore the detection latency distribution is also an underestimate of full-system latency for this reason. With 1, 024 × data reduction, 88% True Positive Rate (TPR) was achieved at h = 3, the latency distribution was 113 ms with 1.86 false-alarms per second.

## 4 Discussion

In contrast to systems such as Chorley et al. (2009) and Kamiyama et al. (2004), and in common with MSOWS (Lo Preti et al., 2022b), this system is not dependent on marker tracking. Instead, it observes the effect of surface deformation on the passage of light through the transparent waveguide.

A comparison with MSOWS is given in Table 2. MSOWS uses a number of infrared emitters (PEs) and detectors (PRs) embedded all around the skin's periphery (24 of each). The PEs are strobed in sequence, the combinations of PEs and PRs define rays across the skin whose precision is limited by emission and reception cones, and the contact is localized by mapping the resulting reductions in received light at each PR to a virtual grid across the skin area (that is, a 16 × 16 grid with 5 mm cell pitch). In contrast, our DVS-based skin uses two 640 × 480 event-driven cameras with lenses, in combination with a number of PEs (32) around the skin periphery. The PEs are constantly illuminated so that events are not produced in static conditions. Therefore, the power cost for illumination is elevated in our system, however, synchronization of strobing is not necessary. The strobing rate places a limit on the temporal precision of MSOWS, which can only be improved by increasing the strobing rate and thus the data rate. The DVS-based system does not have such a restriction, since event cameras only produce events in the case of change. The latency of DVS is known to vary with illumination and can be as low as a few microseconds with good illumination. The experimental scenario and the system presented here do not allow for the potential latency advantage to be demonstrated or evaluated, since a press can be reliably distinguished from background noise after 31 ms.

**Table 2:** Comparison of this work and MSOWS.

**Metric**	**Lo Preti et al. (2022) (MSOWS)**	**This work (DVS-based)**	
		**No data reduction**	**1,024**×**reduction**
Sensor type	Optical waveguide (PE-PR)	DVS (stereo)	
Data reduction	N/A	No data reduction	1024×
Number of view points	24	2	
Processing unit	FPGA DAQ board	On-board event processing	
Samples per press	168 samples/PR	Event stream (sparse)	
ADC bit depth	12-b	21-bit	
Press duration	0.144 s	0.55 s	0.55 s
Bit rate (press)	~145 kBs	~975 kBs	~1 kBs
Bit rate (idle)	~145 kBs	~24 kBs	~0.02 kBs
System latency	—	0.031 s Detection latency distribution	0.113 s Detection latency distribution
Global error	1.8 %	3.75 %	~6.4 %
RMSE (localization)	—	4.66 mm	9.33 mm
Effective num taxels over sensor area	—	105	34
Effective num taxels over probed area	—	46	15

Given static illumination, the lenses are necessary to disambiguate the directions from which presses are detected and offer high precision for localization. In the method presented here, DBSCAN is used to cluster the events corresponding to a contact. The center of the detected cluster is extracted along the horizontal (*u*) axis of each camera and used to determine the angle of incidence based on the camera's field of view, which may, in principle, give an arbitrarily better accuracy.

The characterization of the localization accuracy is given in terms of the distance between the estimated location and the actual press location, with an RMSE of 4.66 mm. To contextualize this, an effective number of taxels can be estimated by calculating how many circles of radius 4.66 mm fit into the sensor area. Across the *probed* region of the sensor, this corresponds to 46 effective taxels (if the same RMSE were to hold across the full sensor area, this would be equivalent to 105 taxels, but we do not claim this, since the RMSE has only been measured within the probed region. It is likely that other effects, such as distortion or the proximity of the LEDs, would be more prominent toward the edges of the sensor).

For a better comparison with MSOWS, the Contact Map Reconstruction Error (CMRE) statistic for global error in a single press scenario (Lo Preti et al., 2022b) has been computed. This system achieves a global error of 3.75 % vs. MSOWS's 1.8 %.

The employed DVS cameras are not optimized for this application and may be highly overspecified. It may be that small pods of DVS pixels in 2 or more locations could deliver more efficient performance. To evaluate the possibility of using fewer event data, events have been randomly eliminated within trials, and the results shown in the Table 2 are for a 1,024 × reduction. In this case, the CMRE rises to 6.4 %, RMSE rises to 9.33 mm, the effective number of taxels reduces to 34 (15 probed), and pressure detection latency distribution is approximately 31 ms and remains at about 113 ms under a 1,024 × data reduction. By applying a 1024 × data reduction, the DVS version can cut its peak press-mode bit-rate from 75 kBs down to 1 kBs, and its idle bit-rate from 24 kBs to 0.02 kBs, compared to MSOWS's constant 145 kBs. MSOWS used 12-bit A-D conversion, whereas we have considered 21 bits per DVS event, given the size of the VGA address space.

In addition to comparing our system with MSOWS, we place it alongside other recent tactile localization technologies. These include resistive and tomographic skins, capacitive arrays, optical tactile sensors, magnetic skins, inductive and microwave sensors, and acoustic approaches. These systems vary widely in materials, wiring complexity, sensing principles, and achievable accuracy, but all aim to localize contact across a surface. The main differences between our DVS-based skin and these technologies concern sensing modality and data rate: our approach uses two event cameras instead of dense arrays, avoids global inverse problem solving, and naturally supports low idle bit-rate operation. A summary comparison of these technologies is provided in Table 3.

**Table 3:** Comparison of representative tactile localization technologies.

**Sensor**	**Localization capabilities**	**Materials**	**Accuracy/ resolution**	**Techniques (hardware/algorithm)**
Electrical Impedance Tomography (EIT) sheet (Chen et al., 2025b)	Multi-touch with bending	Conductive hydrogel; elastomer layer	5.4 mm error	EIT reconstruction with ML for touch–bend decoupling
Hydrogel EIT skin (Hardman et al., 2023)	Multi-touch	Self-healing ionic hydrogel	12.1 mm	Data-driven EIT reconstruction
Stretchable capacitive array (Sarwar et al., 2017)	Multi-touch under stretch/bending	Transparent elastomer; stretchable electrodes	1 cm resolution	Capacitive grid with deformable substrate
Magnetic skin (Hu et al., 2024)	Multi-touch, multi-scale; large-area sensing	Magnetized elastomer film; hall sensors	1.2 mm error over 48,400 mm area	Signal processing with Convolutional Neural Network (CNN)
Graphene Hall magnetic array (Li et al., 2025)	Single-touch with virtual 6 × 6 resolution	Graphene Hall elements; magnetized layer	1.3 mm average error	Vertical periodic magnetization to reduce field interference
Flexible EIT tactile skin (Chen et al., 2025a)	Multi-touch with bending	Magnetic hydrogel layer and Ecoflex	4.8 ± 2.8 mm touch error; bend RMSE ~0.9°	CNN state classifier
SonicBoom (Lee et al., 2025)	Single-touch	MEMS microphones	0.43–2.22 cm localization error	Vibration-based triangulation with learned model

Only single-touch localization has been investigated in this work. Low-pressure detection and multi-touch interactions were not studied. Early tests (not shown here) agree with previous findings in MSOWS that contact can still be detected through internal light reflection when the surface is bent. With the reduced viewing locations provided by focused optics, the present system could also be extended to larger or more complex surfaces. Beyond localization, we did not attempt any further tasks such as identifying the type of contact or classifying the source of the pressure. However, the event data contain richer information than just position. Features such as the shape of the event cluster, the number of events produced, and the timing of the event activity could support future work on force estimation, slip detection, object recognition, or classification of different touch types. These tasks would require additional processing and calibration and remain outside the scope of this proof-of-concept study, but they represent clear next steps.

### 4.1 Limitations and future directions for multi-touch sensing

A key limitation of the current work is its exclusive focus on single-point contact localization. The methodology presented, which relies on identifying a single dominant event cluster in each camera, is fundamentally unequipped to resolve multiple simultaneous touch events. This is a critical consideration for future applications in complex, interactive scenarios.

Another limitation is that the current system does not estimate the magnitude of the applied pressure. This work was designed as a proof of concept focused on press localization. The robot pressed the sensor surface with a cubic tip to a fixed indentation depth of 2 mm; position control was used and the applied force was not measured during the experiments. However, the event data suggest that pressure estimation could be added in future work. As shown in Figures 6C, D, a single press produces a characteristic cluster of events on each camera. By analyzing features such as the size of the activated region, the density of events, or the temporal evolution of the cluster during indentation, it may be possible to infer the applied force. A dedicated calibration procedure would be required to map these features to physical pressure values.

Another limitation of the present work is that the evaluation was carried out only within the central region of the sensor surface. In early tests, presses near the edges of the silicone produced noticeably larger localization errors, as the image distortion increases toward the periphery. More importantly, the silicone layer had to be made very soft (30:1 PDMS, see Section 2.1) to maximize sensitivity, and this made the material mechanically fragile at the edges. Since the NIR LEDs are embedded directly in the silicone along the boundaries, pressing too close to an edge caused the silicone to lift and risk damage to the LEDs or tearing of the material. For these reasons, the robot was constrained to press only within the safer central region of the sensor during the experiments, corresponding to 52.7% of the usable sensing area. A more robust fabrication process, would be needed to allow full-area testing in future work.

The failure arises from the classic stereo correspondence problem. In a multi-touch scenario, each DVS camera would detect multiple, distinct clusters of events. The current algorithm would either erroneously merge nearby clusters into a single, inaccurate centroid or, if modified to detect multiple centroids, would lack a mechanism to correctly pair the corresponding centroids from the left and right cameras. For instance, with two presses generating centroids *L*_1_ and *L*_2_ in the left camera's view and *R*_1_ and *R*_2_ in the right's, the system could not disambiguate between the correct pairings (*L*_1_-*R*_1_, *L*_2_-*R*_2_) and the incorrect “ghost” pairings (*L*_1_-*R*_2_, *L*_2_-*R*_1_), leading to erroneous localization.

However, the event-based nature of the DVS offers a powerful solution. It is highly improbable that two distinct mechanical presses would be initiated in the exact same microsecond. Future work can implement a temporal correlation filter. By segmenting event clusters based on their onset timestamps, the system can pair clusters from the two cameras that appear and evolve within a narrow, shared time window (e.g., ≤ 1 ms), thus robustly solving the correspondence problem for non-synchronous contacts.

An alternative approach is to move beyond simple centroiding and utilize richer spatial features of the event clusters. The shape, size, total event count, and velocity profile of corresponding clusters should be similar across both camera views. A future algorithm could combine epipolar geometry constraints with a feature-matching technique (e.g., comparing cluster moments or event density histograms) to calculate a matching score and identify the most probable pairings.

For guaranteed disambiguation in cluttered or highly dynamic scenarios, the system could be extended with a third, non-collinear camera. This would provide an additional geometric constraint, allowing for robust triangulation even with ambiguous pairings between any two of the views. The intersection point validated by all three viewpoints would confirm a true contact, while 'ghost' points would be rejected for failing this multi-view consistency check.

Beyond the current triangulation-based method, an alternative approach to resolving the stereo correspondence problem in DVS-based tactile sensing is the use of Spiking Neural Networks (SNNs). Osswald et al. (2017) proposed an SNN model that enables 3D perception by leveraging spike-based representations and temporal dynamics.

## 5 Conclusions

This work presents an optical skin system designed for advanced tactile sensing using DVS cameras with a compliant silicone layer. Based on the MSOWS design (Lo Preti et al., 2022b), this work proposes a marker-free event-based tactile sensor by replacing embedded photoreceivers with DVSs cameras. Our approach allows for localization of contacts through stereo vision and triangulation, with a simple, flexible, and responsive design suitable for dynamic tactile applications. The system achieves a pressure localization accuracy of 4.66 mm RMSE. Data reduction analysis reveals that the event-based approach could sustain a pass rate above 85 % even when reducing data transmission by a factor of 1,024, confirming the feasibility of a low-bandwidth implementation. The detection latency distribution is 31 ms, demonstrating the potential system's suitability for real-time tactile interactions.

By utilizing DVS technology, the system benefits from high temporal resolution and reduced data bandwidth, making it well-suited for scenarios requiring real-time processing and high-speed interaction. Furthermore, the system's marker-free design simplifies integration, paving the way for scalable and adaptable soft robotic applications.

## 6 Future work

While the current design is a proof-of-concept that successfully localizes contacts, several areas for improvement remain.

Future work will focus on a new design that grants the optical skin higher sensitivity. The next envisioned steps include extensive testing on various tactile tasks, such as precise localization, slip detection, and force mapping. These would be useful in fine-tuning the system to achieve better accuracy and reliability, and become more easily deployable in soft robots and interactive objects. Unused data features such as V-address, polarity, and precise timing may be used to increase the system's discrimination ability. Optics may be better matched to the employed cameras. Smaller cameras with fewer pixels may provide acceptable performance with even lower data rates. Moving toward a design with small clusters of pixels and dedicated optics introduces key trade-offs. While it reduces the overall data processing load and lowers bandwidth requirements, it increases wiring complexity. Additional synchronization strategies may be required between clusters to maintain localization accuracy. Furthermore, decreasing the number of pixels limits the resolution of event detection, potentially impacting fine-grained spatial accuracy. However, a well-optimized optical system could compensate for these constraints, enabling a balance between data efficiency and sensing performance. SNN-based methods might be investigated as an alternative to triangulation, potentially improving accuracy and resolving remaining systematic skew and adaptability to more complex tactile interactions.

## Data Availability

The dataset used in this study is available at Zenodo: https://zenodo.org/records/17274644. The code for processing and analysis is available on GitHub: https://github.com/event-driven-robotics/optoskin.

## Footnotes

1
https://github.com/event-driven-robotics/optoskin/blob/main/latency/latency_from_rates_main.py
